# Comprehensively analysis of immunophenotyping signature in triple-negative breast cancer patients based on machine learning

**DOI:** 10.3389/fphar.2023.1195864

**Published:** 2023-06-23

**Authors:** Lijuan Tang, Zhe Zhang, Jun Fan, Jing Xu, Jiashen Xiong, Lu Tang, Yan Jiang, Shu Zhang, Gang Zhang, Wentian Luo, Yan Xu

**Affiliations:** Department of Breast and Thyroid Surgery, Daping Hospital, Army Military Medical University, Chongqing, China

**Keywords:** triple-negative breast cancer, immunotherapy, immunophenotype, prognosis, chemotherapy

## Abstract

Immunotherapy is a promising strategy for triple-negative breast cancer (TNBC) patients, however, the overall survival (OS) of 5-years is still not satisfactory. Hence, developing more valuable prognostic signature is urgently needed for clinical practice. This study established and verified an effective risk model based on machine learning methods through a series of publicly available datasets. Furthermore, the correlation between risk signature and chemotherapy drug sensitivity were also performed. The findings showed that comprehensive immune typing is highly effective and accurate in assessing prognosis of TNBC patients. Analysis showed that IL18R1, BTN3A1, CD160, CD226, IL12B, GNLY and PDCD1LG2 are key genes that may affect immune typing of TNBC patients. The risk signature plays a robust ability in prognosis prediction compared with other clinicopathological features in TNBC patients. In addition, the effect of our constructed risk model on immunotherapy response was superior to TIDE results. Finally, high-risk groups were more sensitive to MR-1220, GSK2110183 and temsirolimus, indicating that risk characteristics could predict drug sensitivity in TNBC patients to a certain extent. This study proposes an immunophenotype-based risk assessment model that provides a more accurate prognostic assessment tool for patients with TNBC and also predicts new potential compounds by performing machine learning algorithms.

## Introduction

Breast cancer is one of the most common cancers among women worldwide, which has different pathological and molecular subtypes including luminal A, luminal B, human epidermal growth factor receptor overexpression (HER-2+) and triple-negative breast cancer (TNBC) (Chodosh, 2011). TNBC is a subtype of breast cancer that estrogen receptors (ERs), progesterone receptors (PRs) and HER-2 are absent and accounts for approximately 15%–20% of all breast cancers (Brenton et al., 2005). Notably, TNBC present the worst prognosis and highest mortality compared with other subtypes and has a wide range of genetic, immunophenotypic, morphological and clinical characteristics (Carey et al., 2007; Dent et al., 2007). Only 30%–50% TNBC patients present pathologic complete response (pCR) after given the standard neoadjuvant chemotherapy regime (including taxane and anthracyclines), which is significantly lower than HER-2+ breast cancer (von Minckwitz et al., 2012; Cortazar et al., 2014). Although various treatment strategies have been developed, however, more than 70% TNBC patients present metastasis and recurrence within 3 years after surgical resection, meaning the prognosis is still poor (Sharma, 2016; Huynh et al., 2020).

Immunotherapy for cancer is often based on the cancer immune cycle theory, which includes the enhancement of stimulatory immune factors and immune checkpoint inhibitors (ICIs) (Gao, 2019; Pio et al., 2019; Sanmamed and Chen, 2019; Hegde and Chen, 2020). The successful application of ICIs has been observed in various types of cancers, including melanoma, hepatocellular carcinoma and lung cancer, and this has caused great excitement (Chee et al., 2017; Luke et al., 2017; Llovet et al., 2018). Unfortunately, the clinical benefit of immunotherapy for most TNBC patients is still limited until nowadays. Previous studies have explored classification strategies for cancer immunotyping (Chen et al., 2020; Zhao et al., 2021). The classification strategy based on immune score and infiltration score has been used in lung cancer and urothelial cell carcinoma (Fu et al., 2018; Tan et al., 2020). However, until nowadays, few research has been done to categorize TNBC using these scores, and to further investigate their correlation with TNBC prognosis and drug sensitivity.

Therefore, establishing reliable predictive biomarkers to identify subgroups that may benefit for TNBC is urgently needed. Besides, adopting comprehensive evaluation of tumor immunophenotype-based treatment strategies for each patient through cancer immune-cycle and immune cell infiltration status are essential to promote the development of effective immunotherapies. In the current study, publicly accessible data of TNBC were retrieved from The Cancer Genome Atlas (TCGA) database, Gene Expression Omnibus (GEO) database and Molecular Taxonomy of Breast Cancer International Consortium (METABRIC) database to establish the robust signature through a series of bioinformatics methods. By combining multi-gene expression datasets, we developed and validated the risk model based on cancer immunophenotypes, and explored its performance in predicting prognosis. In addition, we comprehensively investigated the association between this signature with immune-related characteristics, immunotherapy response and drug sensitivity in TNBC patients. Our results suggested that this cancer immunophenotype-based signature could be used as a promising biomarker in predicting clinical outcome and immunotherapy response in TNBC patients.

## Materials and methods

### Data collection and procession

This research was conducted on publicly available database through a series of bioinformatics methods. Transcript profiles and corresponding clinical information of six cohorts containing a total of 694 TNBC patients were acquired to construct and validate the risk signature after removing the samples with unknown survival time and outcome. Details were as follows: microarray dataset GSE103091 (107 samples) was downloaded from Gene Expression Omnibus (GEO, https://www.ncbi.nlm.nih.gov/geo) and was selected as the training cohort on account of the optimal sample size. Another three microarray datasets named GSE16446 (107 samples), GSE20685 (225 samples) and GSE20711 (78 samples) were also obtained from GEO database and used as the validation cohorts, in addition, TNBC RNA sequencing datasets which downloaded separately from The Cancer Genome Atlas (TCGA, https://portal.gdc.cancer.gov) and Molecular Taxonomy of Breast Cancer International Consortium (METABRIC, https://www.cbioportal.org/) were used as another two validation cohorts, respectively. Summary information of above cohorts was listed in Table 1. Besides, three real-world immunotherapy cohorts (GSE91061, GSE135222, IMvigor210) were chosen to verify the ability of the risk signature on prediction of immunotherapy response. All the raw data were normalized and log_2_ transformed.

**TABLE 1:** Distribution of clinical characteristics among two immune subtypes in three datasets.

	Subtype 1	Subtype 2	*p*-value
	(n = 361)	(n = 209)	
**Dataset**			0.000
GSE16446	55(0.15)	52(0.25)	
GSE103091	187(0.51)	51(0.24)	
GSE20685	119(0.33)	106(0.51)	
**Age**			0.895
(26,46)	70(0.41)	62(0.41)	
(46,55)	41(0.24)	32(0.21)	
(55,64)	30(0.18)	30(0.20)	
(64,90)	30(0.18)	24(0.16)	
**Grade**			0.452
Grade1	2(0.04)	(0)	
Grade2	10(0.18)	9(0.17)	
Grade3	38(0.69)	43(0.83)	
**Primary tumor (T)**			0.114
T1	34(0.20)	42(0.26)	
T2	118(0.69)	92(0.58)	
T3	8(0.05)	15(0.09)	
T4	10(0.06)	11(0.07)	
**Regional lymph nodes (N)**			0.997
N0	72(0.42)	66(0.41)	
N1	62(0.36)	59(0.37)	
N2	23(0.13)	22(0.14)	
N3	14(0.08)	14(0.09)	
**Metastasis**			0.999
Non-metastasis	117(0.67)	123(0.67)	
metastasis	58(0.33)	61(0.33)	
**Status**			0.562
Alive	82(0.76)	167(0.78)	
Dead	26(0.24)	45(0.21)	

### Identification of TNBC molecular subtypes

The Tumor Immunophenotype (TIP) database (http://biocc.hrbmu.edu.cn/TIP) is a webtool that can assess the immune microenvironment on the base of the cancer-immunity cycle (Xu et al., 2018). The marker genes were retrieved from the TIP database and employed to classify TNBC patients into diverse clusters in the training cohort through “ConsensusClusterPlus” R package (Wilkerson and Hayes, 2010). Pam algorithm and “spearman” were used as the metric distance. Each bootstrap process including 80% of the training cohort of patients and was repeated by 500 times. The number of clusters was set to be 2 to 10, and the optimal classification was determined by calculating the consistency matrix and consistency cumulative distribution function (CDF).

### Quantification of the infiltration immune cells and immune-related pathways

The stromal score, immune score and estimate score of training cohort were calculated by ESTIMATE algorithm and were used to compare the immune infiltration between different subtypes and different risk models (Danilova et al., 2019). Then, the c2. cp.kegg.v7.5.1 gene set was downloaded from Molecular Signatures Database (MSigDB, https://www.gsea-msigdb.org/) and employed to quantify the pathways through “ssGSEA” method. The infiltration level of 10 immune cells were also quantified through “MCPcounter” algorithm. Next, we also calculated the relative infiltration level of 22 kinds of immune cells by CIBERSORT method. Then, the characteristic genes of 28 immune cells which obtained from previous study (Charoentong et al., 2017) were used to calculate the degree of infiltrating immune cells between different risk groups in TNBC.

### Differential expression analysis and functional enrichment

Differential expression analysis between diverse molecular subtypes and risk models were conducted by “limma” package and visualized through volcano plot. The selection criterion was |log_2_FC| > 2 and *FDR* < 0.05 for molecular subtypes, and |log_2_FC| > 1.5 and *FDR* < 0.05 for diverse risk models, respectively. The “WebGestaltR” package was used to further investigate the functions involved in differential expressed genes (DEGs), and the Gene Ontology (GO) and Kyoto Encyclopedia of Genes and Genomes (KEGG) enrichment analysis were performed. The Gene set enrichment analysis (GSEA) was then conducted to further analysis the difference of biological functions between different groups based on the Hallmark gene set through “clusterProfiler” package.

### Construct risk model based on machine learning

To develop a consensus model with high accuracy and stable performance, we integrated 10 machine learning algorithms and 101 algorithm combinations. The integrative algorithms included random survival forest (RSF), elastic network (Enet), Lasso, Ridge, stepwise Cox, CoxBoost, partial least squares regression for Cox (plsRcox), supervised principal components (SuperPC), generalised boosted regression modelling (GBM), and survival support vector machine (survival-SVM). The signature generation procedure was as follows: (a) Firstly, univariate Cox regression was employed to identify the prognostic genes in training cohort; (b) Then, 101 algorithm combinations were performed on the prognostic genes to fit prediction models based on the leave-one-out cross-validation (LOOCV) framework in the training cohort; (c) All models were detected in five validation datasets (GSE20685, METABRIC, TCGA-TNBC, GSE16446, and GSE20711); (d) For each model, the Harrell’s concordance index (C-index) was calculated across all validation datasets, and the model with the highest average C-index was considered optimal. The risk score was calculated as following formula: $Risk\;score=\sum_{1}^{n}\mathrm{Exp}*coefficient$ .

### Mutational landscape analysis

The “maftool” package was used to explore the somatic mutations in TCGA-TNBC patients and the top 10 mutated genes were presented in waterfall plot. Besides, the copy number variation (CNV) data of TCGA-TNBC was also downloaded and used to display the proportion of deletion and amplification of genes according to the risk model.

### Comparison of risk models with clinical parameters and TIDE performance

In order to explore the superiority of the risk signature, the time-dependent area under curves (tAUC) of the signature and other clinicopathological features were analyzed and compared in METABRIC cohort and TCGA-TNBC cohort, respectively. Then, the TIDE score of three immunotherapy cohorts (GSE91061, GSE135222 and IMvigor210) were calculated through the online tool (http://tide.dfci.harvard.edu/) for immune treatment effect evaluation. The tAUC of risk signature and TIDE score were analyzed in the three cohorts and the comparison between these two indicators were also performed to distinguish the sensitivity and specificity to immunotherapy response.

### Drug sensitivity analysis

To further investigate potential therapeutic target drugs in the high-/low-risk group, we used the drug-sensitive cell lines in the CCLE database (https://portals.broadinstitute.org/ccle) as a training set. The drug sensitivity of each patient in the GSE103091 cohort was predicted by CTRP and PRISM methods. Then screening for potential regulation of drugs through the setting as |cor| > 0.3.

### Statistical analysis

R software (https://www.r-project.org, version 4.1.3) and GraphPad Prism 8.0 (GraphPad Software Inc., San Diego, CA, United States) were used for all statistical analysis and visualization. Univariate Cox regression analysis was performed to evaluate the significant prognostic genes. Quantitative data were compared between different groups through *Wilcoxon rank sum test*. Relationships between risk scores and expression levels of different genes were examined by *Spearman’s* correlation analysis. Unless otherwise specified, *p* < 0.05 was considered as statistically significant.

## Results

### Diagram of the research

**FIGURE 1:**
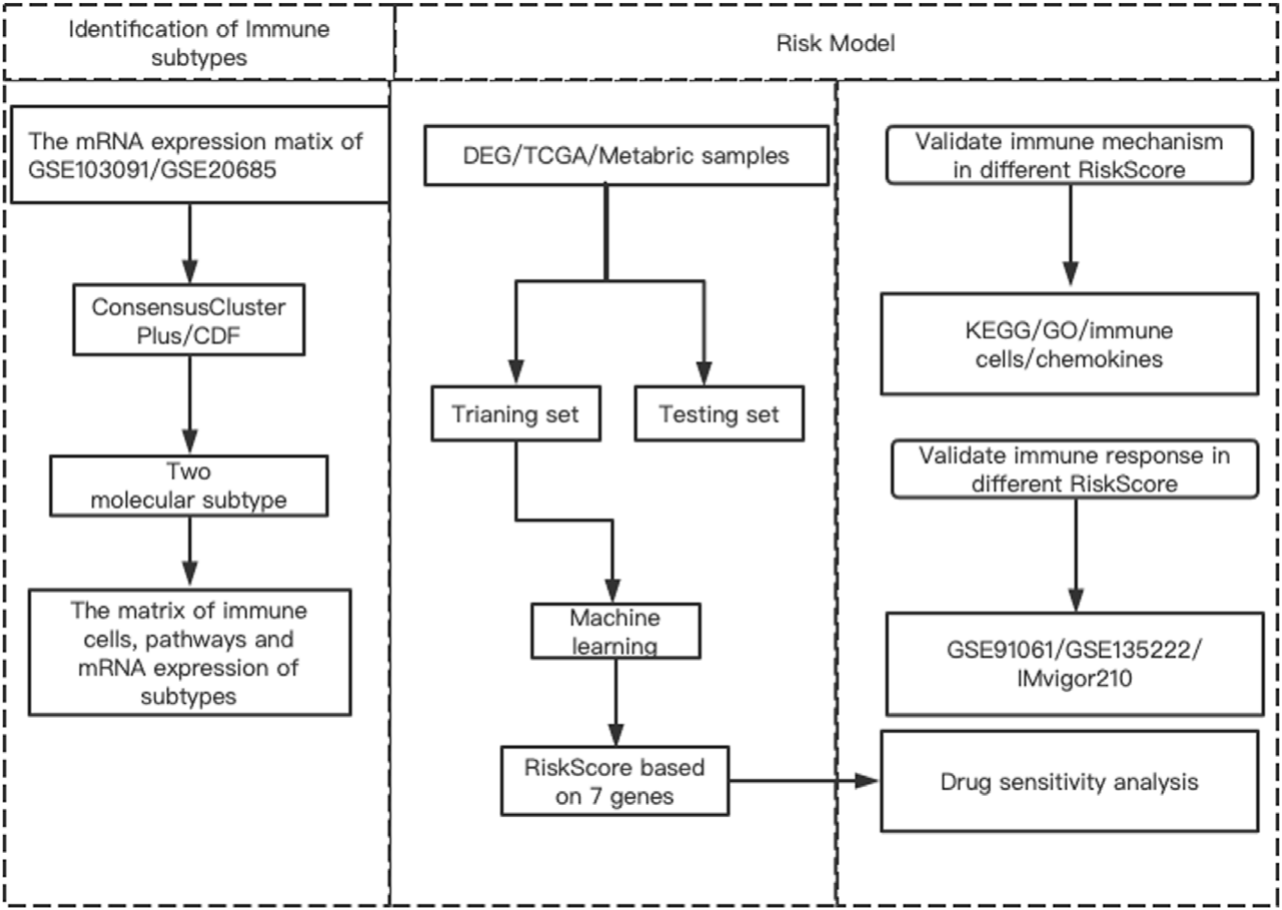
The flowchart of this study.

The workflow of our research was presented in Figure 1. The research contents mainly included three parts: 1) Identifying different prognostic immune types and their related DEGs and pathways; 2) Building risk models based on machine learning methods according to these DEGs and exploring the regulatory pathways of different risk models, as well as the relationship with immune cells and chemokines; 3) Analysis of potential targeting drugs for different risk groups.

### Two diverse molecular subtypes were gathered based on TIP-related genes

**FIGURE 2:**
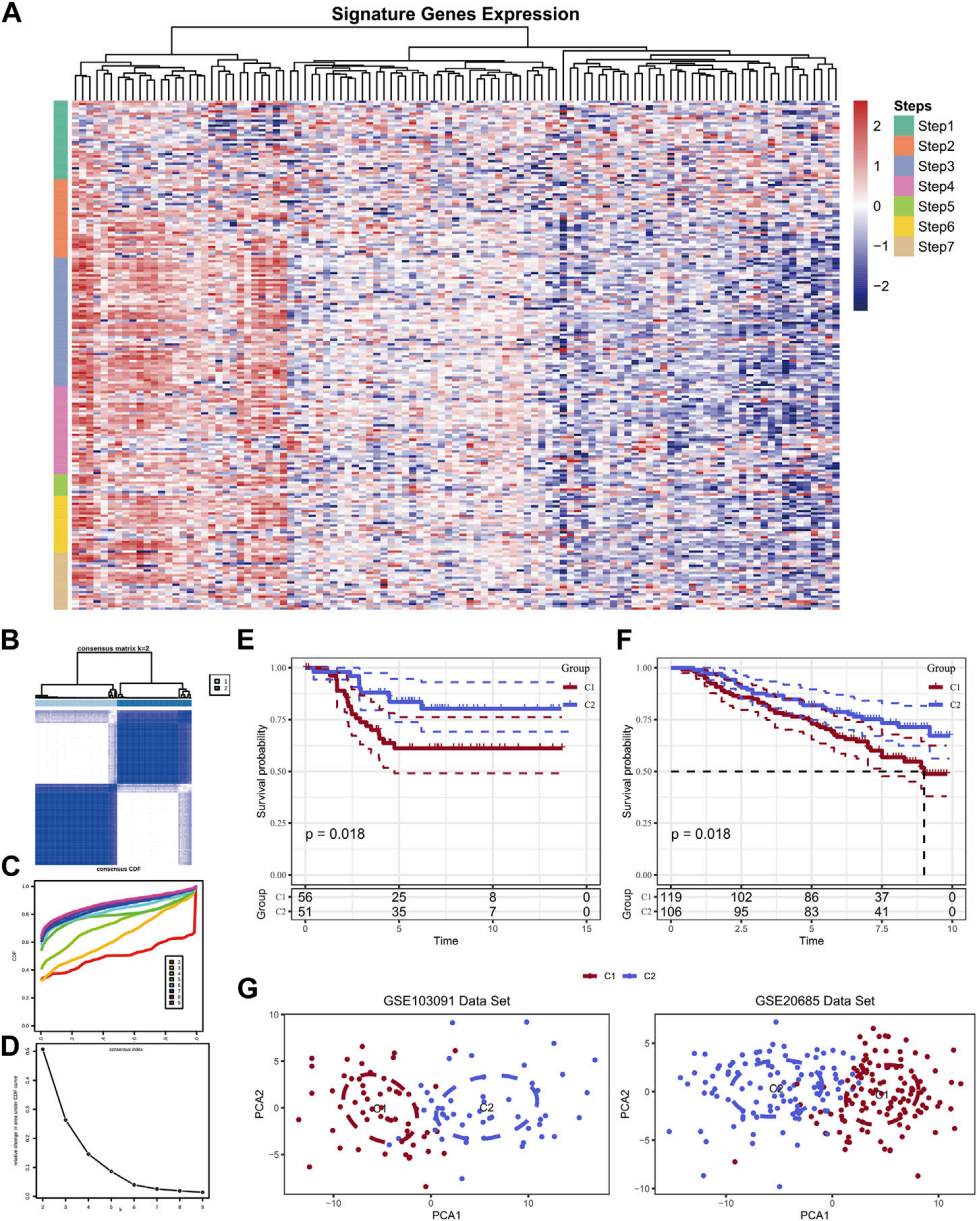
Expression of TIP-related genes in the GSE103091 dataset **(A)**; Sample clustering heat map when consensus k = 2 **(B)**; CDF curve of GSE103091 cohort sample **(C)**; CDF Delta area curve of GSE103091 cohort sample. Delta area curve of consensus clustering, indicating the relative change in area under the cumulative distribution function (CDF) curve for each category number k compared with k-1. The horizontal axis represents the category number k and the vertical axis represents the relative change in area under CDF curve **(D)**; Prognostic relationship between two subtypes of GSE103091 **(E)** and GSE20685 **(F)**; The two data sets were clustered using PCA **(G)**.

A total of 166 marker genes were collected in the seven stages of the cancer-immunity cycle, including checkpoints, cytotoxic factors, chemokines, and major histocompatibility complex (MHC) molecules (Figure 2A). The CDF delta area curve indicated that k = 2 could gather relatively stable clustering results which named Cluster 1 (C1) and Cluster 2 (C2) (Figures 2B–D). Further analysis of the prognostic characteristics of these two molecular subtypes revealed significant overall survival (OS) difference between them in the training cohort (Figure 2E). In general, C1 subtype had a poor prognosis compared with C2 subtype (*p* < 0.05). Similar results were observed in GSE20685 cohort (Figure 2F, *p* < 0.05). Then, PCA analysis was conducted based on Neutrophils marker genes, and PCA dimension reduction distributions of the two subtypes were shown in Figure 2G. The results demonstrated an obvious batch effect between the two cluster samples.

### C2 exhibit relative higher level of immune cell infiltration and immune-related pathway activity

**FIGURE 3:**
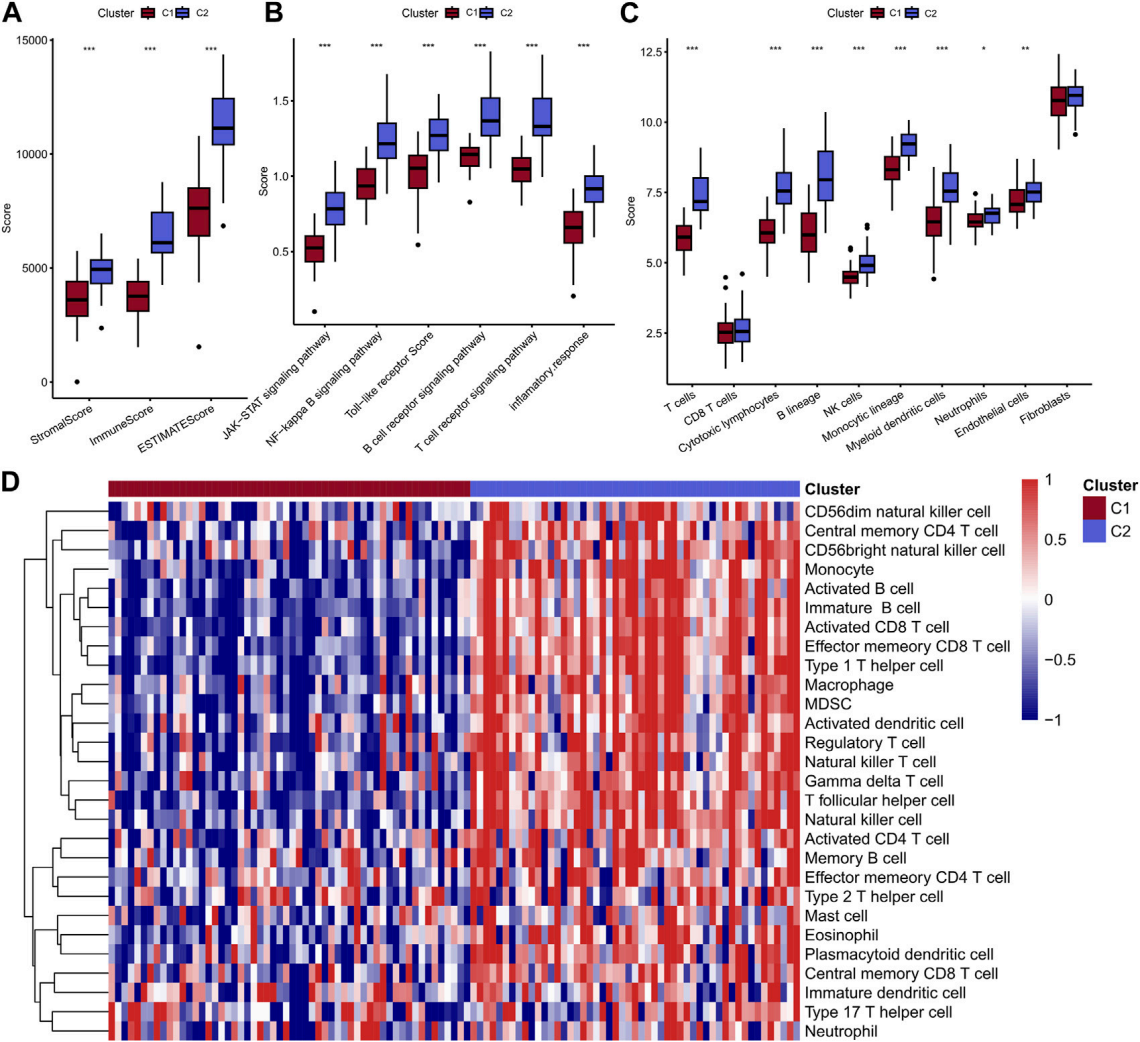
Comparison of immune scores of two molecular subtypes **(A)**; Comparison of scores of 6 inflammatory pathways **(B)**; MCPcounter calculated the abundance scores of 8 immune cells compared with 2 stromal cells **(C)**; ssGSEA calculated the scores of 28 immune cells, and the results were presented in heat maps of the two subtypes **(D)**.

**FIGURE 4:**
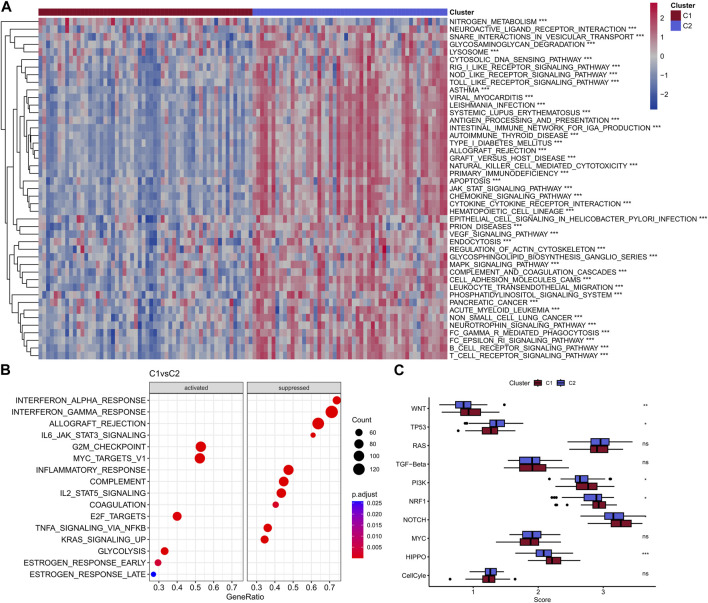
Heat map of enrichment scores of two subtype enrichment pathways in the GSE103091 dataset **(A)**; Bubble map of C1 subtype enrichment to pathway in GSE103091 data set **(B)**; Box plots of ssGSEA scores for 10 tumor-associated pathways **(C)**.

Above results indicated that the patients in C2 showed a better prognosis than the patients in C1. Next, the study explored the differences in immunity between these two clusters. Obviously, C2 displayed a higher immune score, stomal score, and ESTIMATE score compared with the C1 (Figure 3A). In addition, the two clusters showed significant differences in most immune-related pathways, including JAK-STAT signaling pathway, NF-kappa B signaling pathway, Toll-like receptor pathway, B cell receptor signaling pathway, T cell receptor signaling pathway and inflammatory response (Figure 3B). Besides, the quantitative infiltration levels of most immune cells were much higher in C2 than C1, suggesting the patients in C2 may act more immune activity, detailed information were presented in Figures 3C, D. The difference of KEGG pathways were visualized by heatmap and a coincident result was obtained, that is the C2 exhibit higher activity in tumor immunity related pathways, such as apoptosis and JAK-STAT pathway, et al. (Figure 4A). GSEA analysis further suggested the C1 showed positively correlation with cancer-related pathways, including G2M checkpoint and E2F targets, et al., while the C2 presented positively relationship with immune-related pathways, including INF-alpha response and INF-gamma response, et al. (Figure 4B). Finally, the results of “ssGSEA” score showed that five tumor-related pathways were significantly different between two clusters, including WNT, TP53, PI3K, NRF1, and HIPPO, which have been linked to the development and progression of cancer and have great potential in predicting the prognosis of TNBC patients (Figure 4C).

### DEGs between the two diverse clusters showed remarkable functional enrichment in immune-related pathways

**FIGURE 5:**
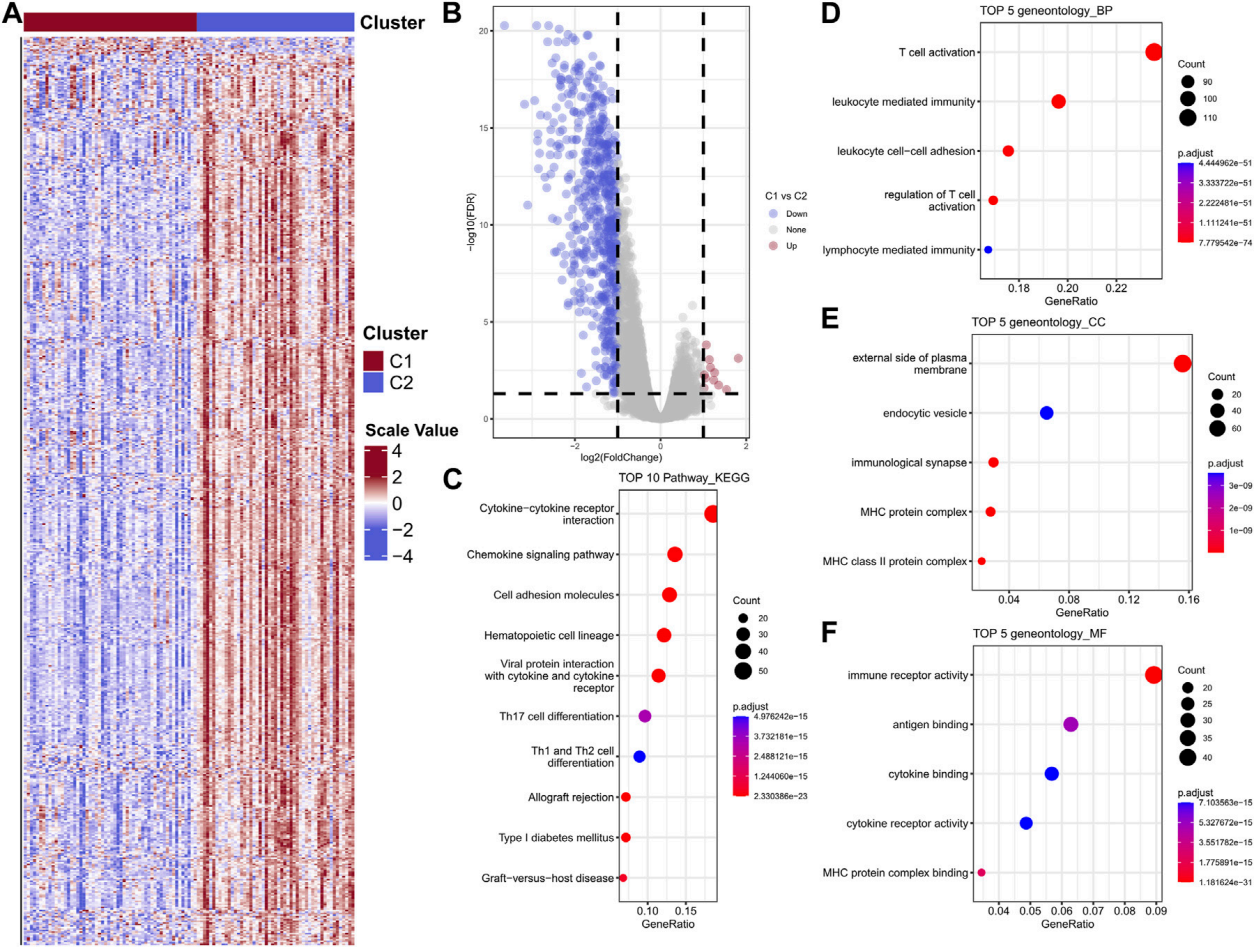
Heat map of differential gene expression between two subtypes in the GSE103091 cohort sample **(A)**; Differential gene volcano map **(B)**; Differential gene enrichment analysis, KEGG, BP, CC, MF **(C–F)**.

In order to further investigate and verify the differences of biological functions between the two clusters, the differential analysis was performed to complete this task. The heatmap was showed in Figure 5A. A total of 590 DEGs were collected, among which contains 11 upregulated genes and 579 downregulated genes (Figure 5B). KEGG enrichment indicated the DEGs were mainly participate cytokine-cytokine receptor pathway, chemokine signaling pathway, cell adhesion molecules, hematopoietic cell lineage and viral protein interaction with cytokine and cytokine receptor (Figure 5C). GO biological process results showed these DEGs were enriched in T cell activation, leukocyte mediated immunity, leukocyte cell-cell adhesion, regulation of T cell activation and lymphocyte mediated immunity (Figure 5D). The top five cellular component (CC) and molecular functions (MF) were showed in Figures 5E, F, respectively.

### Construct the risk model based on machine learning

**FIGURE 6:**
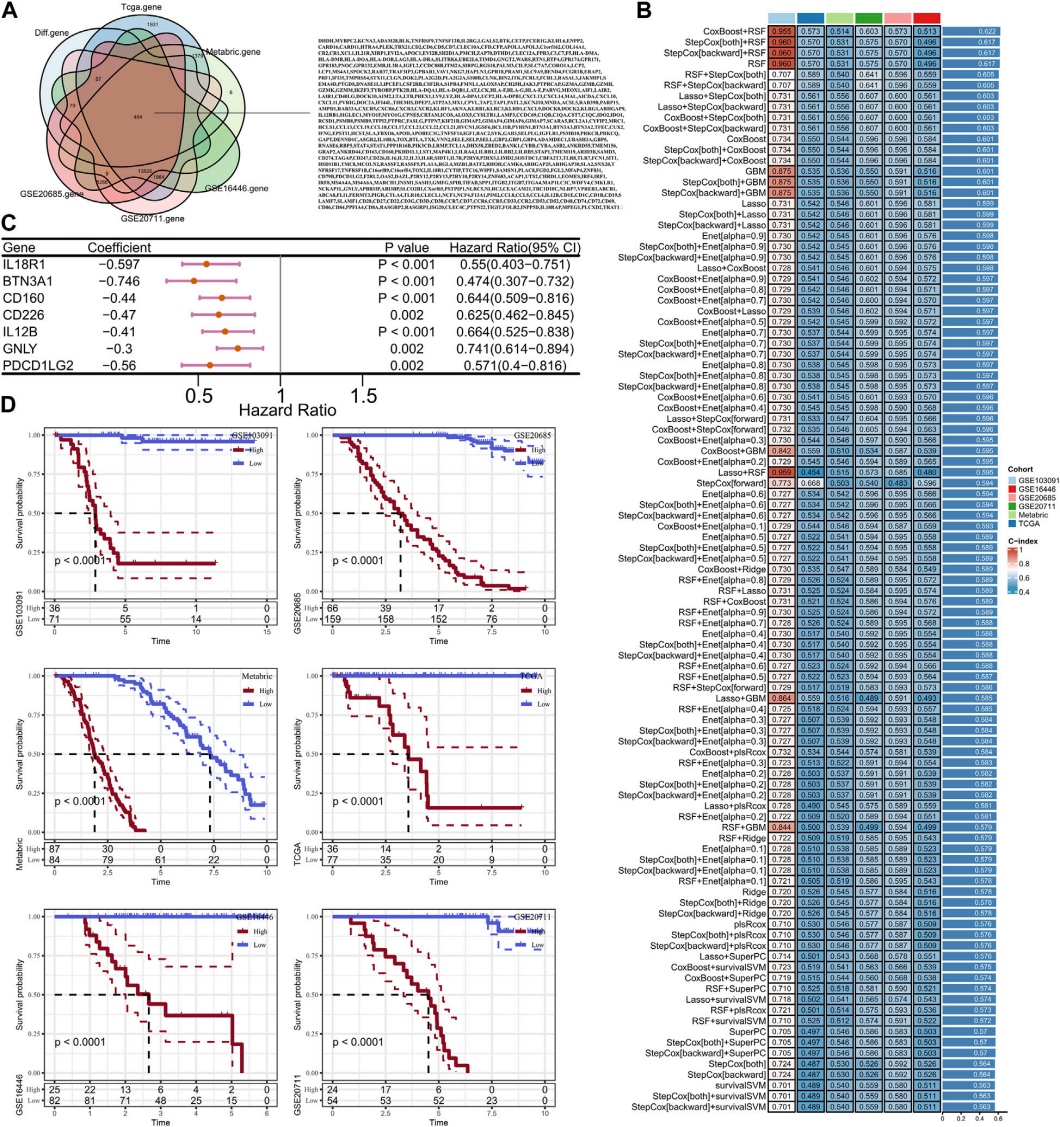
Veen diagram of intersection between GSE103091 differential genes and testing set genes **(A)**; Machine learning screening to construct the optimal combination of risk models **(B)**; Forest map of optimal model-related genes **(C)**; KM curves of high and low risk groups in training set and verification set **(D)**.

A total of 454 genes were collected from the intersection of DEGs, TCGA, METABRIC, GSE20685, GSE20711 and GSE16446 data sets (Figure 6A). Then, univariate Cox analysis was performed to calculate the relationship between 454 DEGs and TNBC prognosis in the training cohort, and 30 prognostic genes were finally screened after filtering *p* < 0.05, among which were all protective factors. Next, these 30 genes were used to develop a consistent prognostic model through our integrated program based on machine learning approach. In brief, 101 prediction models were filtered through the LOOCV framework, meanwhile, the C-index for each model was also calculated in the training cohort and validation cohorts to select the most outstanding candidate. Interestingly, the optimal model was a combination of CoxBoost and RSF, with the highest average C-index equal to 0.622 (Figure 6B). Finally, seven key genes were screened to establish the prognostic signature (Figure 6C). The risk score was calculated as above mentioned and subsequently normalized by the “scale” method. The Z-score equal to zero was selected as the cut-off value to separate the cohorts into high-risk group and low-risk group. Survival analysis indicated that the patients in low-risk group presented significant longer OS compared with the high-risk patients both in the training cohort and the validation cohorts (all *p* < 0.05, Figure 6D). So, this is considered a robust model and worthy to further study.

### Comprehensively analysis of the risk signature and tumor immunity

**FIGURE 7:**
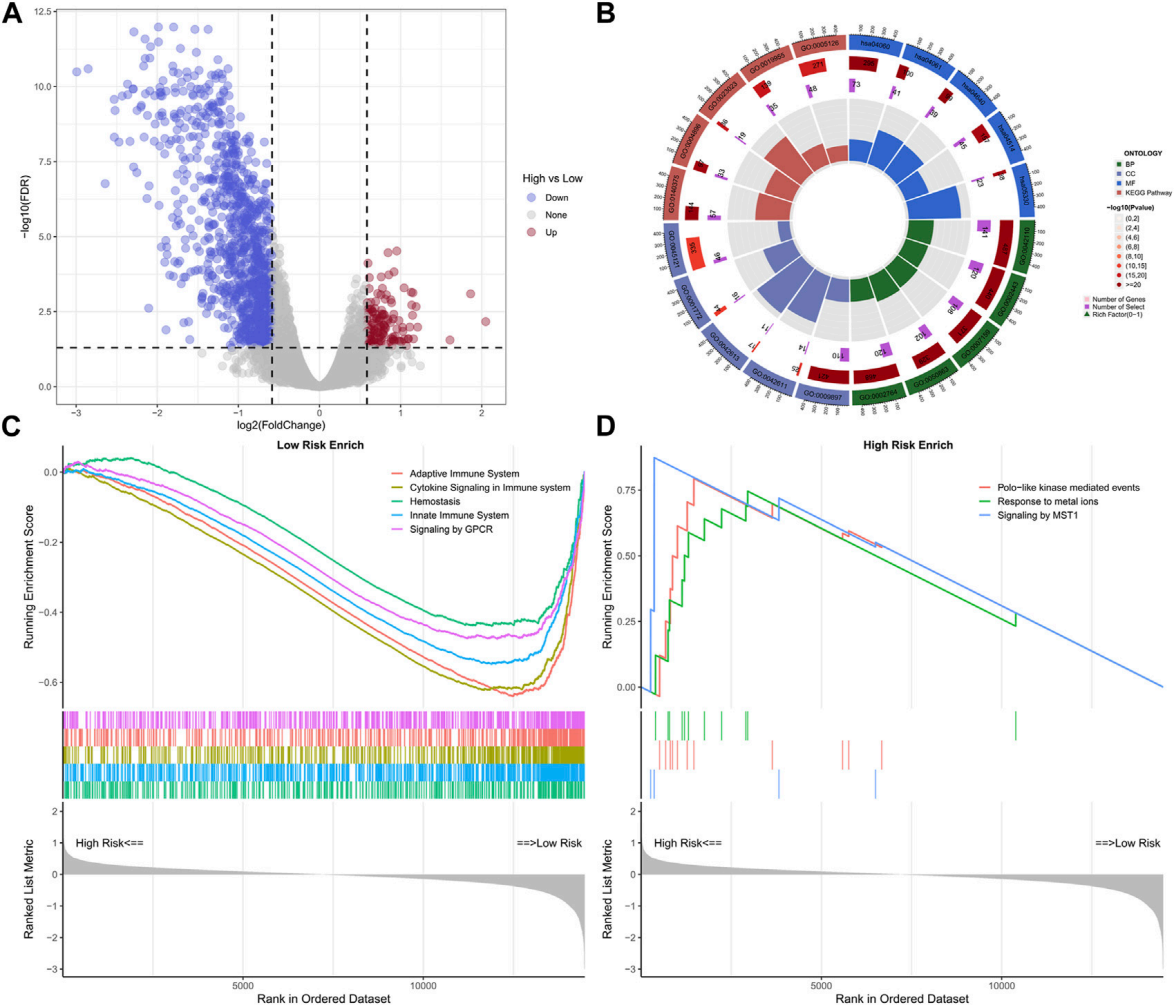
Volcano plot of differential genes in high and low risk groups **(A)**; Loop diagram of enrichment analysis visual display **(B)**; GSEA enrichment analysis of high-low risk group **(C, D)**.

**FIGURE 8:**
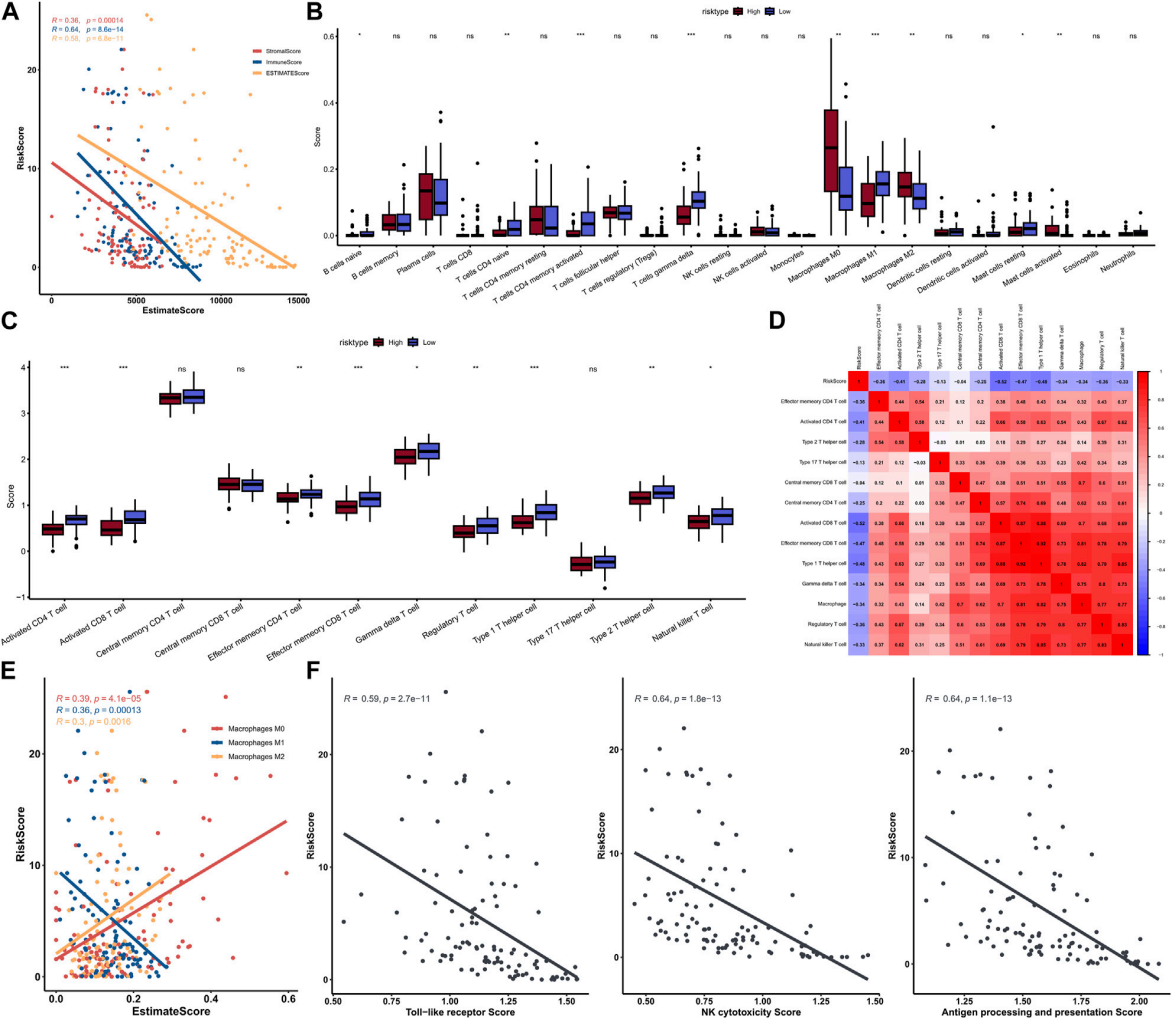
Correlation between risk score and immunity score **(A)**; The scores of immune cells in the high-low risk group in the 22 calculated by CIBERSORT **(B)**; Comparison of T cell scores in high and low risk groups **(C)**; Heat map of correlation between risk score and T cells **(D)**; Correlation graph between three types of macrophages and risk score **(E)**; Correlation between three macrophage-related pathway scores and risk scores **(F)**.

**FIGURE 9:**
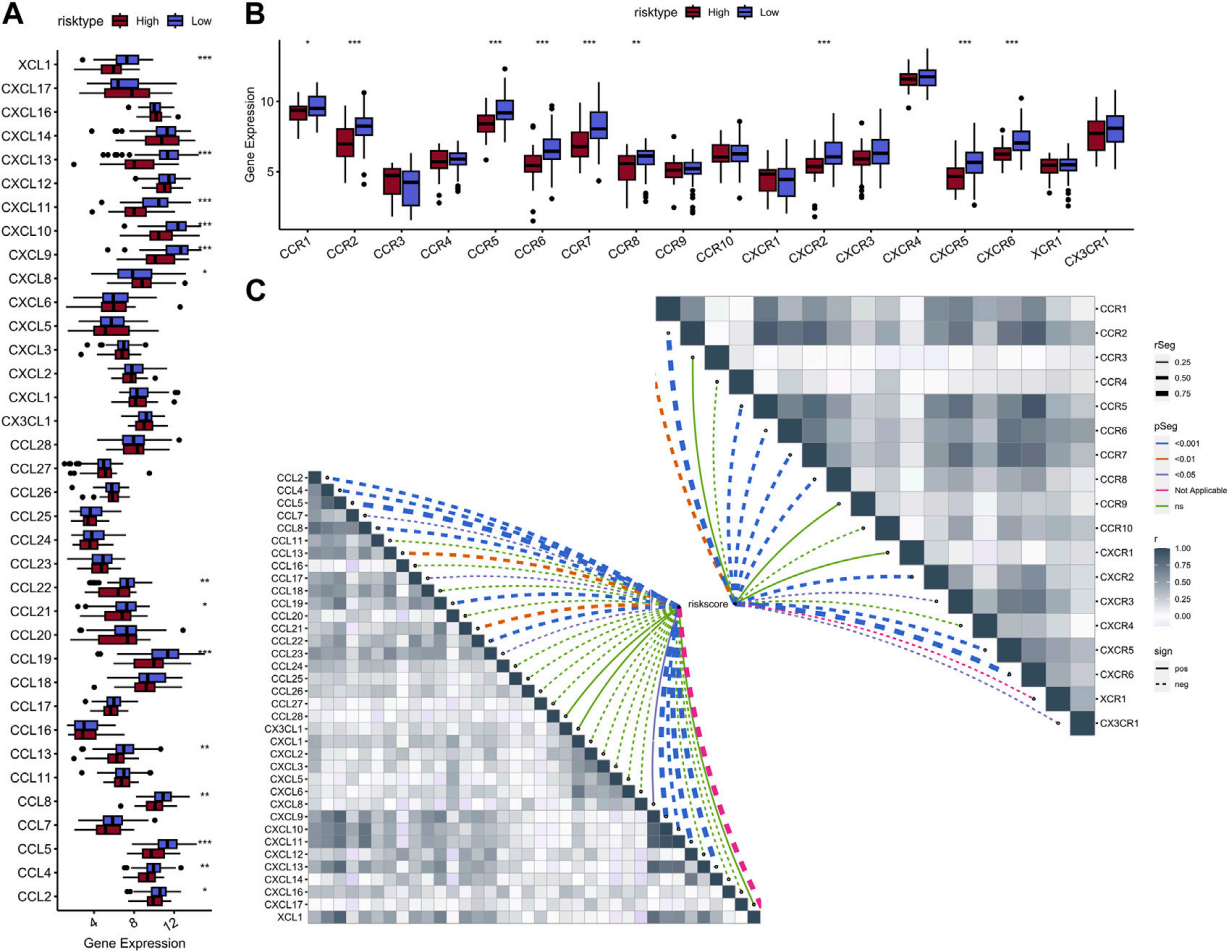
Boxplot of 40 chemokine genes in the high-low risk group **(A)**; Boxplot of 18 chemokine receptor genes expressed in the high-low risk group **(B)**; Heat map of correlation between risk scores and chemokines (bottom left) and chemokine receptors (top right) **(C)**.

A total of 1,145 DEGs were collected between high-risk group and low-risk group in the training cohort (Figure 7A). Functional enrichment analysis indicated these DEGs may play a vital role in immune-related pathways and biological functions (Figure 7B). GSEA results showed that immune-related pathways were significantly enriched in the low-risk group, including innate immune system, adaptive immune system, signaling by GPCR, hemostasis, and cytokine signaling in immune system (Figure 7C). On the other hand, only three pathways were enriched in the high-risk group and most of them were related to cell proliferation process (Figure 7D). Due to the strong correlation between risk characteristics and immune-related biological pathways, we further investigated the association between risk scores and tumor-infiltrating immune cells. Firstly, we use an estimation algorithm to quantify the overall somatic immune cells based on the TCGA sequence. From Figure 8A, we found that the risk score and the immune score presented a strong negative correlation (*p* < 0.001), indicating that the low-risk group which evaluated based on our model had a higher immune infiltration. We further analyzed the differences in the distribution of somatic immune cells between the two subpopulations and found significant differences in T cells and three types of macrophages in the low-risk group (Figure 8B). Then, using the characteristic genes of 28 immune cells obtained from previous study (Charoentong et al., 2017), the infiltration scores of 28 immune cells were calculated by “ssGSEA” method, and 9 out of 12 T cells showed significant differences in the two risk groups (Figure 8C). Furthermore, we analyzed the correlation between risk score and these 12 types of T cells (Figure 8D), and the results showed that there was a strong negative correlation between risk score and T cell scores. It was also found that M1 macrophage score was negatively correlated with risk score, while M0 and M2 showed an opposite trend (Figure 8E). The scores of three macrophage-related pathways were also significantly negatively correlated with risk scores (Figure 8F). It can be seen that 14 out of 40 chemokines were significantly different between the two risk groups, suggesting that different risk groups may have different degrees of immune cells infiltration, and these differences may directly affect the progress of tumor and the effect of immunotherapy (Figure 9A). In addition, we calculated and compared the expression of chemokine receptor genes in the different risk groups and found that there were significant differences in the expression of chemokine receptor genes, including CCR1, CCR2, CCR5, CCR6, CCR7, CCR8, CXCR2, CXCR5 and CXCR6 (Figure 9B). Finally, further analysis indicated the risk score was significant negatively correlated with these genes (Figure 9C). Thus, our study identified and validated two robust immune subtypes based on comprehensively bioinformatics methods.

To further explore the mutational landscape between diverse risk groups, the somatic mutational data was downloaded from TCGA database and used to complete the procession. As a result, top 10 mutated genes were shown in waterfall plot, including TP53, TTN, MUC16, SYNE1, FAT3, SPTA1, CSMD3, DMD, DYNC2H1 and PIK3CA (Supplementary Figure S1A). CNV analysis presented the proportion of deletion and amplification of these seven genes were remarkable changed, especially the CD160 (Supplementary Figure S1B). These findings suggested that these genes with significant mutational differences may play an important role in different immune scores.

### Risk signature performed robust prognostic value and immune response compared with clinical features and TIDE performance

**FIGURE 10:**
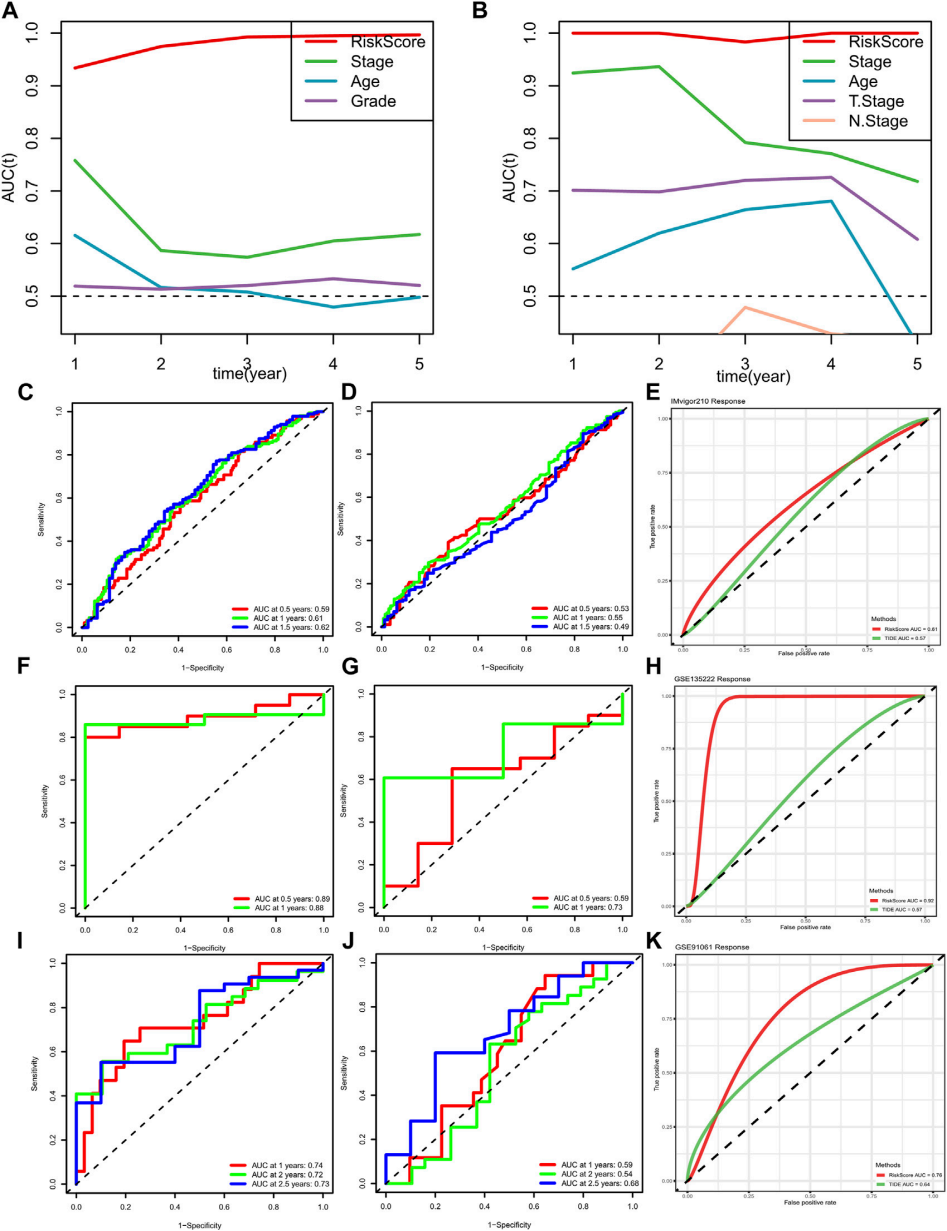
METABRIC and TCGA risk scores compared with AUCs during 1–5 years of clinical characteristics (**A, B**); ROC curve of risk score of GSE91061 dataset **(C)**; ROC curve of TIDE predicted immunotherapy response in data set GSE91061 (**D**); ROC curve of risk score and TIDE effect on immunotherapy in dataset GSE91061 (**E**); ROC curve of risk score of GSE135222 dataset (**F**); ROC curve of TIDE predicted immunotherapy response in data set GSE135222 (**G**); ROC curve of risk score and TIDE response to immunotherapy in dataset GSE135222 (**H**); ROC curve of risk score of data set IMvigor210 (**I**); ROC curve of TIDE predicted immunotherapy response in the data set IMvigor210 (**J**); ROC curve of risk score and TIDE effect on immunotherapy in data set IMvigor210 (**K**).

In order to verify the prognostic performance of risk signature, we conducted tAUC analysis to compare the specificity and sensitivity with other clinicopathological features. Results showed the risk score played a significantly strong survival prediction ability in METABRIC cohort (Figure 10A). Similar results were viewed in the TCGA cohort (Figure 10B). In addition, we calculated the AUC values of the risk model and TIDE score in IMvigor210 cohort, GSE135222 and GSE91061, respectively. Besides, the prognostic value of risk signature and TIDE score were also compared in these three immunotherapy cohorts. All the results indicated the risk signature displayed better ability in prognosis prediction and immunotherapy response (Figures 10C–K).

### Low-risk patients presented higher chemotherapy sensitivity

**FIGURE 11:**
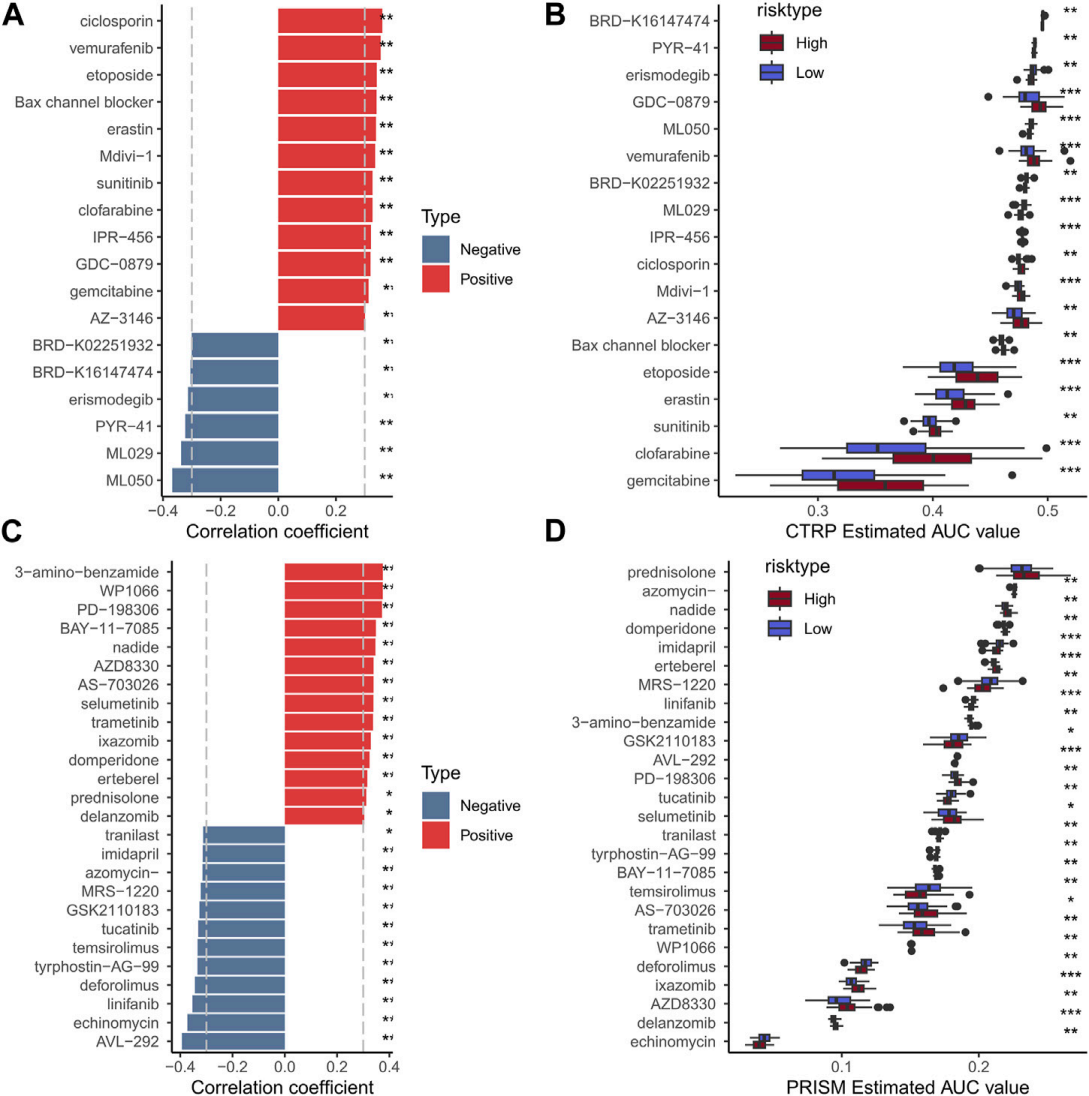
Histogram and boxplot of drug susceptibility predicted by CTRP algorithm and the absolute value of risk score correlation greater than 0.3 (**A, B**); Histogram and boxplot of drugs with absolute value of correlation between drug sensitivity predicted by PRISM algorithm and risk score greater than 0.3 (**C, D**).

To assess the usefulness of risk models in clinical treatment, we analyzed chemotherapy drug sensitivity in low- and high-risk patients. We used the CCLE database of drug-sensitive cell lines as the training set and the GSE103091 data set as the validation set. In the end, a total of 18 CTRP (Figure 11B) and 26 PRISM (Figure 11D) compounds were obtained. The results showed that the high-risk group had higher IC50, indicating that the high-risk group was not sensitive to chemotherapy. Results showed that high-risk groups were more sensitive to MR-1220, GSK2110183 and temsirolimus. Therefore, high-risk samples should be sensitive to these compounds, which may be new options for future TNBC treatment.

## Discussion

TNBC is a subtype of breast cancer with a worst prognosis. However, there is no detailed classification for accurate prognostic assessment and effective treatment at present. Up to now, several studies has developed effectively prognostic assessment based on tumor score and tumor immune cycle characteristics in liver cancer and rectal cancer (Cao et al., 2020; Filho et al., 2021; Hou et al., 2022). Recently, a secondary analysis of a Phase 3 randomized clinical trial showed significant differences in pCR rates among different immunophenotypes during neoadjuvant therapy for TNBC, with higher pCR rates in basal-like and immunomodulatory subgroups. Biological processes associated with basal-like phenotype and immunomodulatory phenotype were analyzed to determine that tumor cell proliferation and immune scores were independent factors associated with the acquisition of pCR. Further validation of immunophenotypes using existing biomarkers may help improve the level of treatment in patients with TNBC (Denkert et al., 2015). CALGB 406036 trail also confirmed a positive association between immune activation and pCR (Meador and Oxnard, 2019). High proliferation and/or immune scores were associated with higher pCR rates when compared with those with low proliferation and/or immune scores. Importantly, immune score was associated with pCR, independent of proliferative score. Therefore, clarifying the immune subtype and providing a precise prediction tool have positive significance for screening the dominant populations of immunotherapy.

Therefore, in the present study, the tumor-infiltrating lymphocyte evaluation was added to develop and validate novel characteristics based on cancer immune cycle for risk stratification, prognosis assessment and drug sensitivity exploration of TNBC patients. Multiple datasets and cluster analysis were used to find the robust immune subtype among TNBC patients. The prognosis was significantly better in the C2 subtype with a higher immune score than the C1 subtype. Thus, predicting immune subtype by a small number of genes expression profiles might contribute to the patient decision of treatment immunotherapies. Recently, a 10-gene lymphoid transcriptomic signature could be used to predict immunotherapy response in human pan-cancer (Ballot et al., 2020). Based on six genes, some researchers had constructed a lung cancer risk score model to provide a reference for individual immunotherapy strategy (Zhang et al., 2021). Some studies constructed the prediction model of immunotherapy response for urothelial carcinoma or lung cancer using deep learning of noninvasive radionics biomarkers (Xu et al., 2019; Park et al., 2020). However, a user-friendly classifier is still not available for TNBC patients. Therefore, the robust signature constructed in the current study will contribute to the clinical implementation of immunotherapy in TNBC.

Immunotherapy was considered one of the effective means for cancer treatment to improve the prognosis of patients (St Paul and Ohashi, 2020; Munari et al., 2021; Li et al., 2022). According to our research, the results demonstrated that C2 showing the higher immune scores, and enriched in T cell, activated CD8 T cells Tleukocyte mediated immunity, leukocyte cell-cell adhesion, which is closely related to T cell activation and lymphocyte mediated immunity regulation. So Patients from C2 would be more likely to be respond to immunotherapy like hot tumors, which had higher levels of T-cell infiltration and some immune checkpoints such as PD-1 and PD-L1 (Galon and Bruni, 2019).

A total of seven key genes were found in our signature, including IL18R1, BTN3A1, CD160, CD226, IL12B, GNLY and PDCD1LG2. As we all know, IL18R1 was expressed on T cytotoxic cells and act as a crucial molecule in the immune microenvironment (Zhang et al., 2020). Its expression level was significantly correlated with stromal, immune, and estimate scores, as well as immune cell levels in lung squamous cell carcinoma (LUSC). The study found that high-IL18R1 and low-IL18R1 groups differed significantly in immune cell composition, including CD8 T cells, NK CD56dim cells, cytotoxic cells, and other immune cells. Moreover, IL18R1 expression was linked with PDCD1, CTLA4, CD8A, and other immune cell markers, highlighting the connection between IL18R1 and the immune microenvironment of LUSC. BTN3A1 was upregulated in TNBC cells and associated with clinical features and immunomodulatory subtype (Poggi and Zocchi, 2014). Interestingly, TNBC patients showed a positive correlation between BTN3A1 and immune cell infiltration. As the primary isoform of the butyrophilin 3A (BTN3A, CD277) family (Zocchi et al., 2017), BTN3A1 directly binds phosphor-antigens, activating the Vγ9Vδ2 T cells in the colorectal cancer microenvironment, generating an anti-tumor response of zoledronate (D'Addio et al., 2013). CD160 played a critical role in bolstering the immune system and was a key member of the CD160/HVEM/LIGHT/BTLA pathway (del Rio et al., 2010). CD160 acts as a costimulatory agent and can be found on multiple immune cells, including intestinal intraepithelial T lymphocytes, CD56dimCD16+ NK lymphocytes, and a minor subset of CD4^+^ and CD8^+^ T cells (Gilfillan et al., 2008). CD226 was a receptor molecule that competing with TIGIT for the same ligands, and has been shown to enhance the cytotoxic and anti-tumor responses of mouse NK cells. Meanwhile, lower levels of CD226+ NK cells have been linked to tumor immune escape (Peng et al., 2016). IL12B variants have been associated with both Crohn’s disease and psoriasis (Cargill et al., 2007). GNLY, encoded by the GNLY gene in chromosome 2p11.2 (Jongstra et al., 1987), has a recombinant 9-kDa form that is cytotoxic to tumors and broadly antimicrobial, killing gram-positive and gram-negative bacteria, yeast, fungi, and parasites (Stenger et al., 1998). PD-L2 has been suggested to play a role in inducing immune tolerance under physiological and pathological conditions (Latchman et al., 2001; Rozali et al., 2012), while also promoting CD8^+^ T cell-mediated anti-tumor immunity (Liu et al., 2003). Higher PD-L1 expression has been observed in TNBCs than non-TNBCs (Muenst et al., 2013; Mittendorf et al., 2014; Muenst et al., 2014), possibly due to genomic amplification of 9p24.1, which contains CD274 (PD-L1) and PDCD1LG2 (PD-L2) in some TNBCs (Barrett et al., 2015). These genes may influence the prognosis of TNBC patients by regulating infiltration of immune cells, such as plasma cells, CD8 cells, M0 macrophages, M1 macrophages, M2 macrophages, and neutrophils. The hub genes identified in the current study play crucial roles in the immune system and constitute a network for determining the prognosis of patients with TNBC.

Comprehensive immune subtyping was developed by immunization scores. The results showed significant prognostic differences between the high and low immune groups. GSEA analysis was performed to explore possible signaling pathways associated with diverse risk groups. These signaling pathways have not been experimentally verified, and further studies are needed to explore the specific mechanisms influencing immune scores in TNBC patients. We further verified the risk model constructed by machine learning. The results showed that a strong negative correlation was found between risk score and immune score, indicating that the low-risk group had higher immune infiltration. Compared with other clinicopathological features, risk score showed strong survival prediction ability, which was very effective and accurate in evaluating the prognosis of TNBC patients. This study suggested the possibility of immunotyping for clinical therapeutic efficacy monitoring, so more TNBC immunotyping data are needed to further support future clinical treatment. But there were several study limitations. Although the evaluation and validation of this risk model across multiple datasets, it remains essential to conduct a large-scale, multicenter, prospective study to authenticate our discoveries. In the time ahead, a series of investigations should be carried out to authenticate the risk model *in vitro* and *in vivo*. As the field of TNBC evolves, it will be important to understand if immune checkpoint inhibitors will improve pCR rates among those patients less likely to respond to standard NAC (e.g., with low proliferation and/or low immune scores).

Considering the application prospect of this model, we further studied the potential therapeutic target drugs in the high-/low-risk group, and screened the potential regulatory drugs according to the drug sensitivity of patients in the data set. New agents and new combinations of immunotherapies may unlock the key to truly personalized cancer medicine. Specifically, efforts focused on understanding biology, biomarker selection, and strategies to enhance immunotherapy response are vital to the success of immunotherapy in TNBC and other cancers in general.

## Conclusion

This study proposed an immunophenotype-based risk assessment model that provide a more accurate prognostic prediction ability for TNBC patients by machine learning algorithms. Meanwhile, new potential compounds which may influence the chemotherapy response were also performed. The disadvantage of this study is that the drug-related conclusions obtained from our research have not been clinically proven at present, and further analysis is still needed to support the study results.

## Data Availability

The original contributions presented in the study are included in the article/Supplementary Material further inquiries can be directed to the corresponding author.
