# Supplementary material for: Comprehensively analysis of immunophenotyping signature in triple-negative breast cancer patients based on machine learning
Source: Front Pharmacol. 2023 Jun 23;14:1195864. doi: 10.3389/fphar.2023.1195864 (PMC10328722; doi:10.3389/fphar.2023.1195864)
Supplement: Supplementary file 1 [file DataSheet1.docx]

Supplementary Material

Article Title

First Author*, Co-Author, Co-Author

*** Correspondence:** Corresponding Author: xy931@163.com

# Supplementary Figure


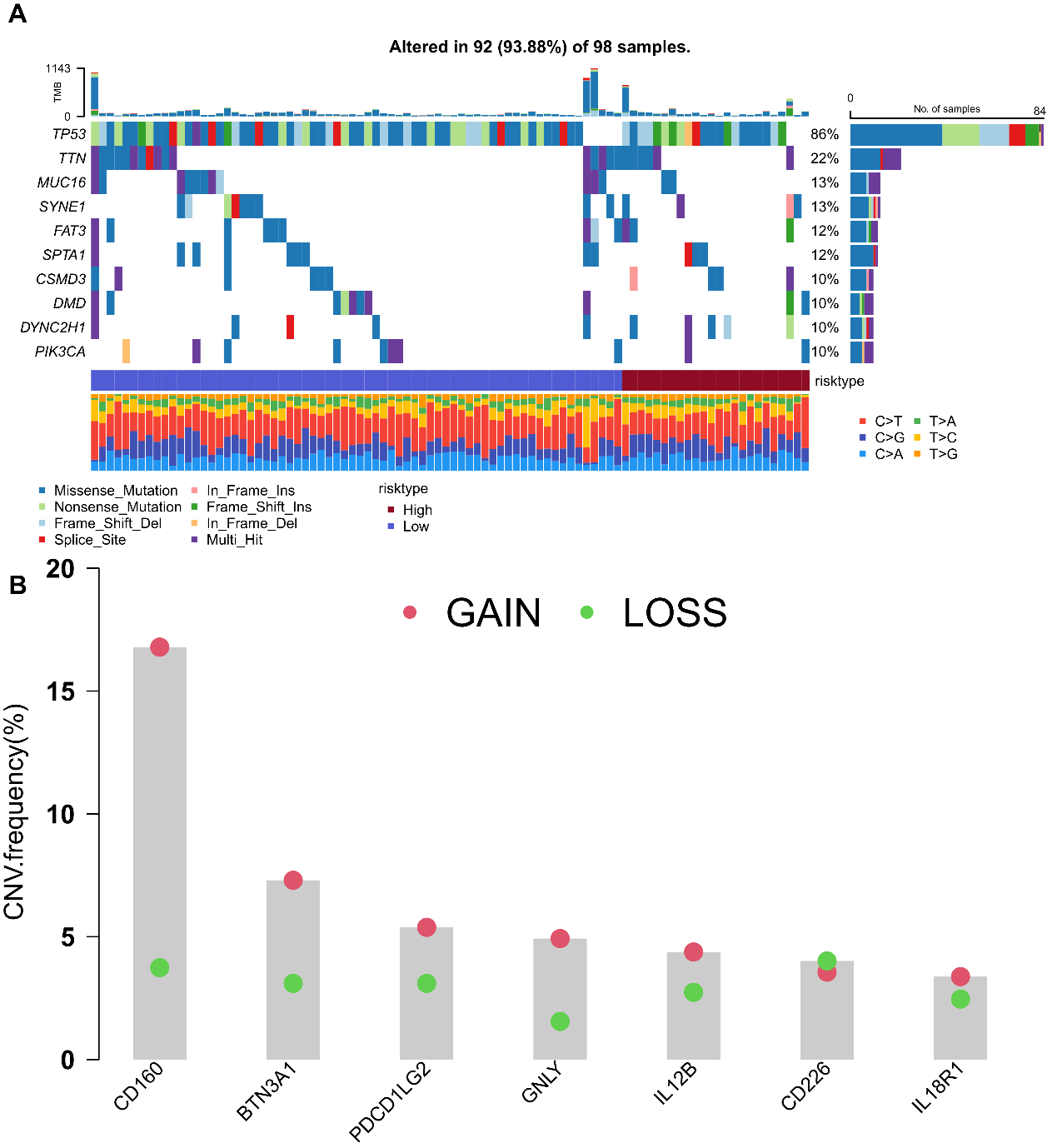


**Supplementary Figure 1.** Genomic changes in high- and low-risk groups in the TCGA cohort. A: Somatic mutation analysis of high- and low-risk groups in the TCGA cohort. B: Proportion distribution of patients with CNV mutation in module gene in TCGA dataset.
